# Focusing in on the Future of Focused Ultrasound as a Translational Tool

**DOI:** 10.3390/brainsci12020158

**Published:** 2022-01-25

**Authors:** Norman M. Spivak, Joseph L. Sanguinetti, Martin M. Monti

**Affiliations:** 1 UCLA—Caltech Medical Scientist Training Program, David Geffen School of Medicine, Los Angeles, CA 90095, USA; 2 Department of Neurosurgery, University of California, Los Angeles, CA 90095, USA; monti@ucla.edu; 3 Department of Psychiatry and Biobehavioral Sciences, University of California, Los Angeles, CA 90095, USA; 4 Department of Psychology, University of Arizona, Tucson, AZ 85721, USA; sanguine@email.arizona.edu; 5 Department of Psychology, University of New Mexico, Albuquerque, NM 87131, USA; 6 Department of Psychology, University of California, Los Angeles, CA 90095, USA

**Keywords:** focused ultrasound, neuromodulation

## Abstract

This article summarizes the field of focused ultrasound for use in neuromodulation and discusses different ways of targeting, delivering, and validating focused ultrasound. A discussion is focused on parameter space and different ongoing theories of ultrasonic neuromodulation. Current and future applications of the technique are discussed.

## 1. Introduction

Ultrasonic neuromodulation was first described in 1957 by Fry, showing that focused ultrasound (FUS) could be employed non-invasively for non-reversible ablation and reversible modulation of biological tissue in both the central and peripheral nervous system [1]. The former (ablative) approach, based on high-intensity transcranial FUS (tFUS) and its mainly thermal effects, has so far seen the most significant development, garnering FDA approval as a non-invasive alternative to surgical interventions for the treatment of essential tremor [2], Parkinson’s Disease [3], benign prostatic hyperplasia [4], prostate cancer [5], uterine fibroids [6], bone metastases [7], and osteoid osteoma [8]. The latter (modulatory) approach, based on low-intensity tFUS and, putatively, mechanical agitation [9], is now (re)emerging as a non-invasive neuromodulatory technique for studying the human brain in both the basic and clinical neuroscientific contexts [10,11,12,13].

While the low-intensity tFUS literature so far has focused primarily on basic and pre-clinical animal science applications, it is undeniable that the ability to target, non-invasively, deep structures of the brain [14] makes it highly promising for clinical applications [15]. Indeed, many neurological and mental health disorders are well known to be associated with dysfunction within deep centers of the brain (e.g., thalamus, ventral striatum, amygdala, globus pallidus) and responsive to surgical neuromodulation (e.g., Deep Brain Stimulation (DBS)). Nonetheless, the cost, eligibility restrictions, and potential complications of surgical interventions reduce their applicability and appeal to patient cohorts, particularly in the context of less severe conditions. Transcranial FUS may, thus, offer an appealing compromise between the ability to target deep brain nuclei, similarly to surgical techniques (e.g., DBS), through a non-invasive approach, similar to transcranial direct current stimulation (tDCS) and transcranial magnetic stimulation (TMS).

Nonetheless, despite a rapid rise in our understanding and exploration of this technique, similar to that in the early days of TMS [16], many factors remain to be discussed, determined, and fully described, particularly in the context of employing tFUS in the treatment of neurological and psychiatric disorders. In what follows, we present an agenda of the principal vital issues that need resolution towards the application of low-intensity tFUS as a clinically applicable tool, and the main strategies currently under investigation to address these issues. We conventionally divide the overview into broad issues, relating to the use of tFUS in general (i.e., including both basic science and clinical applications), and translational issues specific to developing clinical applications.

## 2. General Issues

### 2.1. Standardization

First and foremost, there remains a great need for standardization in the reporting of low intensity tFUS investigations. In part, this is due to the incongruence in which variables are reported. One important variable, for example, relates to measuring the time- and area-varying energy delivered by a specific pulse paradigm. The amount of energy can be described as measured in a water tank—that is, by measuring its diffusion through degassed water (typically referred to as acoustic intensity, I, “point zero”: I.0), or it can be derated (i.e., adjusted) to account for the tissue-specific attenuation as different tissues transmit ultrasonic energies with differing efficiencies (e.g., cerebrospinal fluid, gray matter, white matter, bone). This derated intensity is calculated using the derating equation (I.3 = I.0 * exp(−2αL), where α is the attenuation coefficient, and L is the length of the focus) and is referred to as I.3. Each of these measures conveys different information and allows for different extrapolations, yet much of the literature fails to specify which acoustic intensity is being reported, making it hard to aggregate existing results and interpret any variance across reports, with respect to both effectiveness of stimulation and safety.

**Figure 1:**
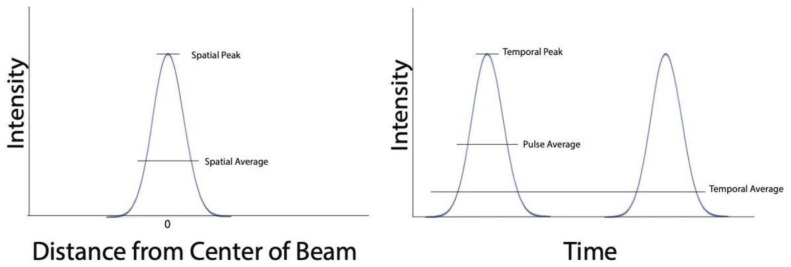
This figure shows how different intensity parameters are measured, in both the spatial and temporal domains.

Furthermore, the acoustic intensity of a sonication depends on the area it is delivered to (spatial-peak versus spatial-average) and on the temporal period (temporal-peak versus temporal-average versus pulse-average) being considered. Considering these variables, intensity can be calculated in several ways, including, in order of decreasing intensity value [17], spatial-peak temporal-peak (I_SPTP_), spatial-peak pulse-average(I_SPPA_), spatial-peak temporal-average(I_SPTA_), spatial-average temporal-peak(I_SATP_), spatial-average pulse-average(I_SAPA_), and spatial-average temporal-average (I_SATA_) (Figure 1). Unless clearly stated, it is not possible for the reader to infer which acoustic intensity is being described.

In 1976, the United States FDA established exposure limits for diagnostic ultrasound (peripheral vascular, 720 mW/cm^2^; cardiac, 430 mW/cm^2^; fetal and other, 94 mW/cm^2^; and ophthalmic, 17 mW/cm^2^; all are I_SPTA.3_) on the basis of the highest intensities being used when the FDA gained regulatory control [18]. However, this is not to imply that these are the safety limits. There have been multiple studies that have gone above this limit and have shown safety above this limit [19,20,21,22].

While one intensity measure is not inherently better than another, the I_SPTA.0_/I_SPTA.3_ and I_SPPA.0_/I_SPPA.3_ are the most reported values, due to safety and regulatory reasons mentioned previously. For maximum repeatability of results, authors should be mindful to specify which intensity is being reported in their manuscripts.

### 2.2. Aiming

#### 2.2.1. CT/MRI-Based Forward Model (K-Wave, Kranion, Sim4Life, etc.)

There exist many different computer software applications for the modeling the attenuation of the ultrasonic energy and visualizing the beam. These applications are designed to incorporate the different attenuation of skin, brain, and skull and model how the skull geometry deforms the ultrasound beam.

One such software is Kranion [23]. Kranion allows users to plan tFUS treatment and estimate thermal rise. It also allows for the post-treatment evaluation of target accuracy. Kranion was developed specifically for use in high-intensity focused ultrasound (HIFU) ablative procedures, using multi-transducer arrays. It can generate metrics of treatment efficacy and efficiency (such as skull density ratio and incident angle). It can model and predict temperature changes at the focus. Indeed, Kranion is an excellent piece of software for use in multi-transducer and multi-element arrays. However, it falls short for single-element experimental setups, such as those used in non-ablative neuromodulatory experiments, which is the focus of this review.

One option that can handle single-element transducers used in low-intensity experiments is the MATLAB package K-wave, which allows for the modeling of photoacoustic wave fields, using a pseudo-spectral time domain method to solve discretized linear wave equations [24]. Using K-wave, it is possible to determine where the focus was based on the transducer location in relation to the subject’s head. It accounts for diffraction of the beam due to individual skull geometry. In fact, K-wave can be used to reverse the wave equations to determine what the best placement of a single-element transducer is for a desired target [25]. This requires imaging to be done in advance of the tFUS session. Thus, K-wave represents a way to both determine the ideal placement of the transducer prior to conducting an experiment, and to determine where the focus of the stimulation if K-wave was not used a priori.

A third software option is Sim4Life. It incorporates a complete model of human anatomy and attenuation coefficients for different underlying tissues (i.e., bone, dura, brain) [26,27]. It can also simulate energy deposition of FUS, utilizing a full-wave acoustic solver based on the finite-difference time-domain method derived from the non-linear Westervelt-Lighthill equation [28]. Furthermore, for those using a single-element transducer, Sim4Life incorporates the hybrid angular spectrum method, which together with the fast near field method, can model inhomogeneous tFUS simulation. Sim4Life is, thus, the most comprehensive software package for ultrasonic modeling available to date. It, however, does not take into account individual subject anatomical variation, and so is not ideal for confirming/validating target placement on a single subject level but, rather, for predicting where the focus will be in different tissues with different ultrasound parameters.

#### 2.2.2. Acoustic Lenses

Lately, much work has been done on the use of custom 3D acoustic lenses to “target” the tFUS beams in low-intensity neuromodulatory experiments [29]. Using the shape of the subjects’ skull and an a priori target, a custom lens can be created to focus the tFUS beam into nearly any shape. One group who developed this technique created a lens that focuses the beam into the shape of a hummingbird, as an example of the power of this approach [30]. Future applications can be imagined in which individualized lenses are created to form the acoustic beam into the shape of virtually any brain structure, such as the amygdala, which can then be focally targeted for anxiolytic treatment [31]. To implement this technique, however, an accurate representation of the skull is required. Traditionally, this meant using CT imaging. However, much work has been done using ultrashort echo MR (e.g., PETRA) imaging, and it remains to be seen whether CT is necessary or if PETRA will be sufficient. It would certainly be revolutionary to the entire field if custom lenses for neuromodulation could be made without the need for ionizing radiation from CT scans.

### 2.3. Neuronavigation

#### 2.3.1. Stereotactic Approaches (Brainsight, Visor2, Soterix)

There are several approaches to target low intensity tFUS using MR guidance. Each method involves acquiring individual structural MRI images to guide the placement of the ultrasound transducer on the participant’s head, which can be done either inside or outside the MRI scanner. One common targeting technique is the “anatomic method”, wherein alignment is performed inside the MRI scanner by placing fixed fiducials markers (e.g., Vitamin E capsules) on the ultrasound transducer (if none are already embedded by the transducer manufacturer) that can be imaged by the scanner. Rapid structural MRI sequences are collected, and, using the ruler tool on the MRI console, lines are drawn from the fiducials that are orthogonal to the transducer face. Since the transducers currently employed have a fixed focal depth, this method will “point” the transducer to the desired region of interest (ROI). This “pointing” approach has been used by multiple studies [15,32,33]. The method is carried out prior to any subsequent MRI sequences. Participants may move their head slightly during the MRI scan, which could cause the transducer angle to change, slightly changing the angle of entry of the focused acoustic beam. As such, one major limitation of the anatomic method is that the researchers will need to perform the aligning method again by collecting another short structural scan at the end of the study to ensure the focus did not change, or, ideally, after each MRI sequence, in order to ensure proper alignment. Thus, it is not a true “real-time” neuronavigation.

MR-guided frameless stereotaxy using an optical tracking system (i.e., BrainSight or Visor2) allows for the “pointing” approach to be applied outside of the MR scanner and in real-time. These “neuronavigation” systems were initially developed to guide transcranial magnetic stimulation (TMS), and have been adapted for ultrasound targeting. Many researchers have employed this method in human studies [34,35,36]. Neuronavigation requires a structural MRI for each participant, which is loaded into the navigation software (BrainSight or Visor2). In the neuronavigation software, a target is placed in the desired ROI on the structural MRI, and the system either generates an automatic straight line of shortest distance to the scalp (Visor2), or the researcher can draw a line from the center of the ROI to any location on the scalp (Brainsight). The scalp location is then used to guide the placement of the transducer on the participants head. Fiducial markers are placed on the participants MRI, and the participant wears reflective markers on their head that are tracked by the cameras. The participants is then coregistered to their MRI by tapping the fiducial markers on their head, which then allows the tracking system to track their head in real-time. The ultrasound transducer also contains markers that enables tracking while it is on the participants head, and the location of the transducer relative to the scalp can be tracked along the *x*-, *y*-, and *z*-axes such that the exact angle of entry can be guided and monitored.

Current neuronavigation systems are designed for TMS applications, and they lack many features that are needed for tFUS research. For example, neuronavigation systems use electric field models to determine the optimal scalp placement relative to the selected ROI. This is feasible because magnetic fields can travel unimpeded by the skull. However, since the acoustic beam of modern tFUS systems is fixed at a particular depth, tFUS systems can target the same ROI from multiple entry points in the skull. Nevertheless, not all entry points are created equal when it comes to tFUS. Areas where the skull bone is thinner (beneath the eyebrow (ophthalmic window), on the temple (temporal window), and the back of the head (occipital window)) are preferred regions for the delivery of transcranial ultrasound. Companies should work with tFUS researchers to update their neuronavigation systems to better meet the strengths of tFUS for more accurate targeting. Another issue is that optical-based navigation must be currently done outside of the MRI scanner, meaning real-time brain changes cannot be monitored. Real-time TMS navigation inside of an MRI has been demonstrated [37], and combining neuronavigation with real-time MRI would be a major advance for the tFUS field.

Neuronavigation systems have a few advantages over the anatomic method. Neuronavigation systems can mark the exact location of the transducer during each pulse of ultrasound by taking a trigger output from the ultrasound system. Thus, researchers can measure the precise location of the ultrasound transducer throughout the entire experiment and quantify the amount of time the transducer was “on target.” Another advantage is that neuronavigation allows for the use of tFUS in conjunction with other recording or stimulating technologies not easily adapted for use inside the MR scanner, such as EEG or TMS. Furthermore, delivering tFUS outside the MR scanner makes it easier to administer treatments across multiple session with the same structural scan, which can save on costs of treatment administration and may be important for clinical applications. Neuronavigation can be performed with an averaged MRI (e.g., MNI152), which may allow for the administration of tFUS to subjects in distant locations who do not have access to an MR scanner facility with tFUS capabilities, or for whom regular visits to a MR scanner are not feasible. It should be noted, however, that there are no empirical comparisons of the target accuracy of using an averaged MRI for navigation for tFUS.

A general issue with all the current targeting approaches is difficulty in assessing where and how intensely the ultrasonic energy is being delivered because they do not account for aberration and attenuation. Typically, the in situ focal zone will be broader due to skull distortion, and shallower due to attenuation [38]. This means that, although accurate targeting at the scalp location is possible, it is impossible to confirm whether the focused beam deposited acoustic energy at the ROI. BOLD responses in the fMRI scans should be used to confirm targeting if tFUS is being performing inside the MRI scanner (see below). For an experiment conducted outside of the scanner, an ideal situation would be to integrate beam models (see Section 2.2.1) into the navigation software, such that they can model the acoustic beam in real time. The advancement of MR-based forward models would significantly accelerate this possibility.

#### 2.3.2. Biomarker Approaches (BOLD, ASL, EEG)

If tFUS is delivered inside an MRI, changes in blood oxygenation level dependent (BOLD) imaging or arterial spin labeling (ASL) can be assessed. These imaging modalities detect changes in neural activity, either through a change in the ratio of deoxyhemoglobin to oxyhemoglobin in the case of BOLD, or through changes in cerebral blood flow in ASL. This approach is an indirect targeting method because the peak signal change may or may not be in the in situ focal zone. It is possible that the tFUS will have its greatest effect downstream in functionally connected regions.

tFUS can also be delivered during simultaneous EEG recording. EEG has inherently much better temporal resolution than BOLD fMRI (1 ms versus 700 ms), and changes in network connectivity can be much more readily assessed with EEG than with fMRI. Concurrent tFUS-EEG has a major advantage over electro-magnetic methods, such as TMS, since ultrasound produces very few artifacts in the EEG [39], lending itself nicely to closed-loop stimulation systems [40]. However, EEG is not the best tool for detecting subcortical change, so any tFUS studies targeting deeper targets should rely on fMRI instead. Furthermore, quality placement of the transducer against the skull requires removal of electrodes in the area of transducer placement, which limits the ability to do EEG source localization.

#### 2.3.3. Visualization Approaches (ARFI/Elastography, Contemporaneous TCD, Thermometry)

Transcranial doppler (TCD) can be used to identify the main arteries in the brain (i.e., Anterior Cerebral, Middle Cerebral, and Posterior Cerebral Arteries; ACA, MCA, and PCA, respectively) [41]. Neuroanatomical structures, such as the hippocampus, lie proximal to the PCA; thus, imaging the PCA with TCD allows one to stimulate hippocampus without the need for expensive MRI machines. This approach only works for targets with well-defined vasculature (i.e., hippocampus) and would not be sufficient for deep brain structures in watershed areas with arborized vasculature (i.e., thalamus).

MR thermometry allows for the measurement of changes to tissue directly caused by tFUS [42]. As the intensity of tFUS increases, so does the temperature of the underlying neural tissue. MR thermometry relies on the fact that the T1 and T2 relaxation times of water depend on temperature. Formulas exist allowing for the calculation of temperature change based on the change in phase, PRF, echo time, gyromagnetic ratio, and magnetic field strength [43]. This approach allows for the precise visualization of the stimulated target, already accounting for the fact that the in-situ focus will be broader and shallower than is estimated using the “pointing” method. MR thermometry is, therefore, well-suited to assess temperature change in ablative tFUS applications, such as in the treatment of Parkinson’s or essential tremor. However, in low-intensity neuromodulatory applications, the temperature change is typically below the detection threshold of the technique, so MR thermometry is not readily of use for these purposes. Rather, it is primarily employed in high intensity applications.

Lastly, MR-ARFI uses the inherent property of tFUS being propagated as a longitudinal wave. As the waves get closer and closer to the focal zone, the waves interfere and lead to an increase in the wave amplitude. Thus, the focus will have the greatest wave amplitude, and the greatest displacement. MR-ARFI uses motion encoding gradients that are applied simultaneously with the onset of the tFUS pulse to image the peak displacement and, thus, the focus [44]. As in MR thermometry, it accounts for the broadening and shallowing of the focus that the “pointing” method does not account for. Lately, much work has been done to improve the sensitivity of this approach, allowing for use at low intensities [45]. The next step would be to establish that k-Wave or Sim4Life are as accurate at visualizing the focus as MR-ARFI.

### 2.4. Stimulation

#### 2.4.1. Parameter Space

Focused ultrasound is described by several variables. The most important is the fundamental frequency. Ongoing studies use a wide variety of values for the fundamental frequency (FF), ranging from 200 kHz all the way to 4 MHz. The value of the FF is what affects the penetration efficacy of the focused ultrasound. It is known that the greater the FF, the less the ultrasound beam can penetrate. Indeed, at 120 kHz, attenuation was fairly mild at around 22% [46]. However, values of FF that are too low can result in overpenetration, leading to standing waves and hemorrhage [47]. Thus, it is important to choose a middle ground frequency that will have sufficient penetration to the anatomic target without overpenetrating and bouncing back.

There is also the pulse repetition frequency (PRF). It is important to note that the FF and the PRF bear no relationship to each other. It is believed by some that the PRF is the main determinant of whether low-intensity tFUS will be excitatory or inhibitory, with PRF values above a specific threshold being excitatory and below that threshold being inhibitory [48]. Others argue the exact opposite, that PRF values below a threshold are excitatory and, above that same threshold, are inhibitory [49].

If the PRF represents how frequently the pulses are delivered, then the duty cycle (DC) represents how much of the time between pulses that sonication is actively delivered. The DC is a percentage, where a 100% DC is continuous (not pulsed) ultrasound. Much research has been done into the effects of the DC on the efficacy of the stimulation. In one study, authors found that the most effective excitatory paradigm used a DC of 70% and the most effective inhibitory paradigm used 3–5% DC [22]. This study used change in somatosensory evoked potentials as a marker for inhibition/excitation. Additionally, yet, other studies used 5% as excitatory [32].

The existing literature has used a variety of targets, PRFs, DCs, pulse widths, and intensities. As a result, comparison is not straightforward. Furthermore, there is unlikely to be a one-size fits all approach, as different neural populations will require different parameters to effect the same signal response. For example, values that are excitatory in thalamus might very well be inhibitory on cortex. As such, there is a great need for further systematic exploration of parameter space. In order to maximize the utility of reported studies, researchers should report at the very minimum, the center frequency, PRF, pulse duration, duty cycle, Pr.0, Pr.3, I_SPTA.0_, I_SPTA.3_, I_SPPA.0_, I_SPPA.3_, focal depth, beam width at focal distance, peak negative pressure, and mechanical index (MI).

#### 2.4.2. Mechanisms

Low-intensity tFUS is thought to modulate neural activity via mechanical stretching of the soma, depolarizing cell membranes by-way-of voltage-gated ion channels leading to increased activity [13]. Many ion channels have been shown to be influenced by ultrasonic stimulation, including mechanosensitive two-pore-domain potassium channels [50], as well as channels not typically classified as mechanosensitive (i.e., sodium and calcium voltage-gated channels) [51]. High-intensity focused ultrasound (HIFU), which is used primarily for non-invasive ablative neurosurgery, relies on a thermal mechanism of action. However, the low intensities and brief stimulation periods used in tFUS do not lead to rises in temperature of more than a degree or so [33,52]. This suggests that tFUS for neuromodulation is not likely to rely on a thermal mechanism, as some have suggested [53].

Another leading theory of mechanism of action is the neuronal-intermembrane cavitation excitation or “NICE” model [54]. This theory proposes that ultrasound causes spaces to expand and contract between the hydrophobic tails of the phospholipids comprising the cell bilayer. By the law of conservation of charge, the greater distance between the charges (inside and outside the cell) results in a greater electric potential, which can change the membrane potential. Whether this change is potentiating (akin to tDCS) or directly modulating (akin to TMS) is yet unclear. More work is desperately needed in the exploration of the mechanistic effects. Part of the issue in determining the mechanism is that, once known, the mechanism will guide parameter selection. Without the mechanism, we do not even know which parameters are truly “excitatory” and which are “disruptive”. One such experiment could be a replication of the Hodgkin-Huxley experiment, to see how ultrasound affects neuronal voltage and ion channels [55].

### 2.5. Safety

#### 2.5.1. Single Target (Fixed/Movable Focus, Single/Multi-Element) vs. Multiple Target

The BrainSonix tFUS system comprises of a single drive computer and a set of single-element transducers [56]. The transducers have fixed focal lengths of 55, 65, and 80 mm, suitable for most applications. The transducers are MR conditional, allowing for the simultaneous collection of fMRI BOLD or ASL data. There are other systems available for commercial use, as well, such as NeuroFUS, Sonic Concepts, and Blatek. These devices are available in MR-conditional variants, as well, and, thus, can potentially be used with simultaneous fMRI or EEG data collection.

Single target tFUS can easily be delivered with a single element transducer. However, with HIFU applications, many targets are needed for a successful treatment. For these applications, single element transducers are insufficient, and more complicated and expensive multi-transducer arrays (such as the Insightec ExAblate) are needed.

#### 2.5.2. Adverse Events

There have been some adverse events reported with low-intensity tFUS. Legon et al., did a retrospective analysis of adverse effects of several of their studies, and published the findings [57]. The most common symptom was sleepiness, reported by 15 out of 64 subjects. However, it was noted that subjects reported it as either unrelated or unlikely in all 15 reports. It is worth noting that the authors found a strong and significant correlation (r = 0.797, *p* = 0.0319) between the I_SPPA_ and the reporting of adverse effects in the 7 individuals who reported mild/moderate symptoms that they believed had “possible” or “probable” cause of tFUS stimulation.

Logically, it makes sense that higher intensities would increase the incidence of adverse events. For this reason, it is even more important to gain a better understanding of how to excite and disrupt the brain with focused ultrasound, so that the lowest possible effective intensity can be used, as this should minimize adverse events.

### 2.6. Interfield Comparison

Low- intensity tFUS could be the “holy grail” of brain stimulation, in that it combines both the spatial accuracy and depth afforded by deep brain stimulation (DBS), without the drawback of having implanted electrodes [16]. At the same time, tFUS is non-invasive, similar to TMS, but allows for the stimulation of much deeper targets than TMS. Thus, tFUS can non-invasively neuromodulate the same neuroanatomical structures as DBS.

Indeed, there is ongoing work with tFUS leveraging prior work done with DBS and TMS. For example, a clinical trial of DBS of central thalamus for disorders of consciousness is now being replicated using tFUS [32,58,59]. Other DBS studies that could be repeated with tFUS include stimulation of basal ganglia for Parkinson’s [60] and entorhinal cortex for memory [61], among others.

With respect to the number of users of tFUS as compared to other brain stimulation techniques, there are many TMS systems with FDA approval (e.g., Apollo, Brainsway, CloudTMS, Magstim, MagVenture, Neurostar, Nexstim, etc.) [62]. These systems are in use by physicians and medical centers all throughout the United States and around the world. Furthermore, nearly every major medical center does DBS surgeries. In contrast, there are only a handful of clinical tFUS researchers throughout the world.

As TMS frequently requires booster sessions to maintain symptom remission and relief, it is likely that tFUS will likely also require booster sessions. This is a purely theoretical conjecture, as there is no evidence to support durability of tFUS effects, and no reason to think tFUS effects will remain permanent. It is possible that booster sessions will not be needed for tFUS, but there is no reason to believe that at this time.

### 2.7. Usability

An important consideration when using tFUS is ensuring good transducer contact with the skin. Inherently, ultrasound travels poorly through air, and these effects get worse as the fundamental frequency increases [63]. Ensuring good contact allows for maximum transmission of the ultrasonic energies. For this reason, hair must frequently be shaved where the transducer will be placed. This is especially important for high-intensity thermal ablation applications, as the trapped microbubbles in hair will absorb energy and cause heating and burns [64]. However, the necessity of head shaving is reduced drastically with lower fundamental frequencies, such that head shaving is likely not needed for frequencies below 0.5 MHz [65]. This is important to note, as female healthy volunteers are unlikely to be willing to have short haircuts, leading to heavily male-biased samples in studies [66]. As discussed previously, 0.5 MHz is roughly in the middle zone of ideal fundamental frequency. Therefore, carefully choosing the fundamental frequency can mitigate the need for subjects to have short hair. This should alleviate the need for predominantly male subject pools.

### 2.8. Noise Masking/Sham/Black Gel Pads

A large issue in conducting double blind studies with low-intensity tFUS is having an effective sham control. This is because active sonication produces not only an audible noise but also significant sound by way of bone conduction. Thus, it is important to have adequate noise masking. One group was able to achieve noise masking using a sound file played through earbuds that decreased the subjects’ ability to distinguish active from sham to below chance levels [67]. This same group also showed that the bone conduction is frequency dependent on the PRF.

It will be important to have a well-designed sham control for clinical and research applications. Part of the utility of tFUS is that it can target deep structures that are implicated in psychiatric disorders (i.e., amygdala and anxiety conditions). Nonetheless, the placebo response in anxiety is extremely high (up to 67% placebo response in generalized anxiety) [68]. As such, it is important to develop an adequate sham control for tFUS.

Since the main noticeable effect is the audible signal produced by the device, a suitable sham control may be as simple as using non-conducting gel pads. Since gel pads are required to facilitate good contact of the transducer, using a specially designed gel pad that absorbs all incident ultrasonic energy would allow for the subject to hear the device and believe they are receiving tFUS when in fact they are not.

An alternative approach would be to use an unrelated brain region as an active placebo. Stimulating this region would give away similar information to the subject regarding whether they are receiving stimulation, but the energy would not be delivered to the region of interest presently under examination. For example, a study of fear and anxiety could use the entorhinal cortex as its sham.

## 3. Single Target vs. Circuit Intervention

Low-intensity tFUS may be delivered to many deep brain structures, such as thalamus, amygdala, and striatum, just to name a few. When using TMS to treat major depressive disorder, the most common target is the dorsolateral prefrontal cortex (DLPFC), which is believed to be hypoactive in depressed persons. The DLPFC is functionally connected to the subgenual cingulate cortex, which is hyperactive in depressed persons, but is far too deep and medial to be reached with TMS. As such, tFUS may be an appropriate tool to target this region. Indeed, some work has been done, targeting this region in conjunction with exosomes to treat refractory depression [69]. So, while both TMS and tFUS are used to modulate nodes of functional circuits, TMS is more suited for modulation of cortical nodes, while tFUS is better suited for modulation of sub-cortical nodes.

A potential treatment for major depression could pair TMS with tFUS, utilizing simultaneous administration of TMS to the DLPFC with administration of tFUS to the subgenual cingulate. By activating the circuit from two sides, perhaps depression could go into remission sooner. TMS and tFUS use different forms of energy (magnetic pulses versus ultrasonic pulses), so there is no reason to expect that the techniques would have any sort of interference effect. However, while there is no fundamental physical interaction between the two techniques, there is the issue of lack of real estate on the scalp surface. TMS coils are large and take away potential areas of transducer placement. For this reason, any such simultaneous stimulation experiment would likely require stimulation of opposite sides of the brain. Furthermore, specialized arms would likely be needed to hold both the TMS coil and tFUS device, as typical head straps would likely interfere with TMS and tFUS placement.

Likewise, there exists evidence of transcranial direct current stimulation (tDCS) being used to stimulate the thalamocortical circuitry in patients in a disorder of consciousness [70]. tFUS could potentially be used to stimulate the thalamus, while tDCS stimulates the cortex. The energies are also of inherently different nature (ultrasonic versus electric) and would not interact with each other.

As disease definitions in neurology and psychiatry move away from single region definitions into circuit-based disease models [71,72,73,74,75], therapeutic modalities will need to be able to modulate all regions of the circuit. Historically, this has only been possible with deep brain stimulation. Here is where tFUS can make its biggest mark.

## 4. Conclusions

There have been many experiments with tFUS since the first experiments were done by the Fry brothers in the 1950s. Since then, both high- and low-intensity tFUS has benefited from the widespread use of MRI and MR guidance. However, with the recent explosion of focused ultrasound, both at high and low intensities, many issues have come to the forefront, and these must be addressed if the scientific community is going to continue to use this very powerful neuromodulatory approach. First and foremost, authors should be mindful to report all stimulation parameters. Second, researchers should choose a neuronavigation approach that is best suited to their experimental design, taking into account concerns regarding confirmation and validation. Third, clinical researchers should be sure to incorporate a blinding effect in low-intensity non-ablative studies to maximize the usefulness of the resultant data and help determine efficacy of the technique. Researchers should also consider their choice of parameters, as this can affect both the necessity of short hair and maximum ultrasound exposure time.
